# Plasma Biomarkers for Monitoring Brain Pathophysiology in *FMR1* Premutation Carriers

**DOI:** 10.3389/fnmol.2016.00071

**Published:** 2016-08-12

**Authors:** Cecilia Giulivi, Eleonora Napoli, Flora Tassone, Julian Halmai, Randi Hagerman

**Affiliations:** ^1^Department of Molecular Biosciences, School of Veterinary Medicine, University of California, Davis, Davis, CAUSA; ^2^Medical Investigation of Neurodevelopmental Disorders Institute, University of California, Davis, Davis, CAUSA; ^3^Department of Biochemistry and Molecular Medicine, University of California, Davis, Davis, CAUSA; ^4^Department of Pediatrics, University of California Davis Medical Center, Sacramento, CAUSA

**Keywords:** Fragile X, metabolomics, mitochondrial dysfunction, neurodegeneration, trinucleotide repeat disease

## Abstract

Premutation carriers have a 55–200 CGG expansion in the fragile X mental retardation 1 (*FMR1*) gene. Currently, 1.5 million individuals are affected in the United States, and carriers are at risk of developing the late-onset neurodegenerative disorder Fragile X-associated tremor ataxia syndrome (FXTAS). Limited efforts have been made to develop new methods for improved early patient monitoring, treatment response, and disease progression. To this end, plasma metabolomic phenotyping was obtained for 23 premutation carriers and 16 age- and sex-matched controls. Three biomarkers, phenylethylamine normalized by either aconitate or isocitrate and oleamide normalized by isocitrate, exhibited excellent model performance. The lower phenylethylamine and oleamide plasma levels in carriers may indicate, respectively, incipient nigrostriatal degeneration and higher incidence of substance abuse, anxiety and sleep disturbances. Higher levels of citrate, isocitrate, aconitate, and lactate may reflect deficits in both bioenergetics and neurotransmitter metabolism (Glu, GABA). This study lays important groundwork by defining the potential utility of plasma metabolic profiling to monitor brain pathophysiology in carriers before and during the progression of FXTAS, treatment efficacy and evaluation of side effects.

## Introduction

A modestly expanded CGG nucleotide repeats (55–200) in the 5′ UTR of the fragile X mental retardation gene, *FMR1* (Kogan et al., 2008; Tassone et al., 2012; Battistella et al., 2013), is the hallmark of premutation carriers. Originally, premutation carriers were thought to be free of phenotypic traits; however, the findings regarding fragile X-associated primary ovarian insufficiency [FXPOI; (Cronister et al., 1991)], followed by the discovery of fragile X-associated tremor/ataxia syndrome (FXTAS; OMIM:300623) identified in adult carriers (Hagerman et al., 2001) discredited this notion. Premutation carriers may also suffer from psychological problems, visuo-spatial deficits, and immune dysregulation (Tassone et al., 2012; Winarni et al., 2012; Wong et al., 2012; Battistella et al., 2013; Hagerman and Hagerman, 2013), while affected children are often diagnosed with ADHD, autism, anxiety, and other psychopathologies (Farzin et al., 2006; Chonchaiya et al., 2013). At the cellular level, fibroblasts from premutation carriers (humans or animal models of the premutation) are generally accompanied by high *FMR1* gene expression, normal or lower levels of its translation product FMRP (fragile X mental retardation protein) and mitochondrial dysfunction (Ross-Inta et al., 2010; Napoli et al., 2011).

It is currently unknown which carriers of the premutation (over 1.5 million women and men in the United States) will develop FXTAS, as clinical diagnosis fails to identify carriers before significant neurological symptoms are evident. Therefore there is an immediate need for early detection and effective drugs for the cure or the prevention of FXTAS.

An understanding of the molecular characteristics underlying disease processes is a prerequisite for the development of early detection biomarkers to provide high value therapeutics. Metabolic profiling is one of the most important techniques, particularly focused on the detection of biomarkers for diagnosis of diseases (German et al., 2005) including metabolic disorders (Wang et al., 2005), motor neuron disease (Rozen et al., 2005), Parkinson’s disease (Scherzer et al., 2007), and Alzheimer’s disease (Barba et al., 2008). Metabolomics, an omics-level analysis of all metabolites, has become one of the main choices for obtaining an overall view of *in vivo* disease states, which has been successfully employed to identify metabolites associated not only with disease, but also with toxicity and aging (Lawton et al., 2008; Boudonck et al., 2009a,b; Sreekumar et al., 2009). Ante-mortem studies generally target body fluids, such as cerebrospinal fluid (CSF), urine, saliva or plasma/serum, as they are thought to reflect changes in tissues. Thus, these non-invasively available biofluids can be used as resources for first screening, in early detection of any disease.

To our knowledge, no analysis comparing biomarkers for differentiating premutation carriers (and their endophenotypes) from controls has been reported to date. Here a global plasma profiling has been developed for the discovery of novel plasma biomarkers applied to the premutation with and without FXTAS. Plasma metabolomics was evaluated in premutation carriers and age- and sex- matched controls with the aim of providing early biomarkers with the potential of uncovering metabolic perturbations in pathways (Goodacre et al., 2004; Werner et al., 2008) associated with the presence of the *FMR1* premutation.

## Materials and Methods

### Characteristics of the Subjects Enrolled in this Study

The study was conducted at the MIND Institute and approved by the IRB ethics committee at UC Davis Medical Center. Exclusion criteria were refusal of the patient or his guardian, infection, or malignancy. Blood samples were obtained by venipuncture with informed consent, and the experiments were undertaken with the understanding and written consent of each subject. Controls and carriers of the premutation were recruited through the Fragile X Treatment and Research Center at the MIND Institute at University of California, Davis, and who participated in our genotype–phenotype study of families with fragile X between the years 2013 and 2015. Blood draws were all performed at the MIND Institute between the hours 8 and 10 am (fasting was not advised). No exercise has been reported by any of the subject before the blood draw (unlikely event since they had to be at the Clinic by 7–8 am on the day of the exam). Clinical evaluations with Dr. Hagerman and associates were performed after the blood draw. CGG repeat number in all individuals was measured using Southern Blot and PCR analysis as previously described (Tassone et al., 2000). The study group named “control” consisted of 16 individuals, seven male and nine females, with an average age (mean ± SD) of 36 ± 13 years and 31 ± 5 CGG repeats (**Table 1**). The group named “premutation” included 23 premutation carriers, 11 men and 12 women, with an average age of 37 ± 19 years. No significant differences in terms of age (*p* = 0.867) or sex (χ^2^ test *p* = 0.804) were observed between these groups except for the CGG repeats (average of longest allele only: 103 ± 40; *p* = 2 × 10^-8^). Four of these subjects were diagnosed with FXTAS utilizing the criteria reported by Jacquemont et al. (2003) (**Table 1**). The average CGG repeats of the mutant allele in heterozygous carriers (females only) was 86 ± 23 (mean ± SD) whereas that of hemizygous carriers (males only) was significantly longer (121 ± 47; *p* = 0.033).

**Table 1:** Demographics and clinical characteristics of plasma donors included in this study.

Subjects	Age (y)	CGG repeats	Sex	FXTAS stage
C1	29	30	M	0
C2	54	30	M	0
C3	23	29, 30	F	0
C4*	50.5	21	M	0
C5	24	30	M	0
C6	41.2	43	M	0
C7	28.8	20, 33	F	0
C8	26	30	M	0
C9	33.7	23, 30	F	0
C10	54	25, 33	F	0
C11	25	24, 33	F	0
C12	45	22, 33	F	0
C13	24	23, 35	F	0
C14	26.3	30, 37	F	0
C15	41.5	20	M	0
C16	57.4	23, 30	F	0
P1	46.3	61	M	0
P2	9.7	31, 63	F	0
P3*	8.4	180	M	0
P4*	24	31, 93	F	0
P5	19.7	177	M	0
P6	55.6	104	M	0
P7	49.3	31, 86	F	0
P8*	17.3	16, 67	F	0
P9	45.3	69	M	0
P10	49.9	20, 98	F	0
P11	9.1	160	M	0
P12	55.4	30, 69	F	0
P13	53	16, 67	F	0
P14	33.1	30, 137	F	0
P15	43.2	30, 106	F	0
P16	38.4	33, 60	F	0
P17*	8.4	180	M	0
P18	24	30, 79	F	0
P19	25	67	M	0
P20*	62.5	105	M	4
P21	61.3	96	M	4
P22	61.8	110–130	M	1
P23	59.1	33, 107	F	3

C and P refer to controls and carriers of the premutation, respectively. The number following the letter identifies the subject from which the fibroblasts were obtained.

*Receiving SSRIs.

### Plasma Metabolomics

Plasma samples were isolated from a single blood draw per individual as previously described (Napoli et al., 2016b) Samples were extracted and analyzed by mass spectrometry as described in detail elsewhere (Napoli et al., 2015). Briefly, 30-μl aliquots were extracted by 1 ml of degassed acetonitrile:isopropanol:water (3:3:2, V/V/V) at -20°C, centrifuged and decanted with subsequent evaporation of the solvent to complete dryness. A clean-up step with acetonitrile/water (1:1) removed membrane lipids and triglycerides. The cleaned extract was aliquoted into two equal portions and the supernatant was dried down again. Internal standards C08–C30 FAMEs are added and the samples were derivatized by methoxyamine hydrochloride in pyridine and subsequently by *N*-methyl-*N*-trimethylsilyltrifluoroacetamide for trimethylsilylation of acidic protons. Data were acquired using the following chromatographic parameters, with more details to be found in (Fiehn et al., 2008; Napoli et al., 2015). Metabolites were identified by matching the ion chromatographic retention index, accurate mass, and mass spectral fragmentation signatures with reference library entries created from authentic standard metabolites under the identical analytical procedure as the experimental samples.

### Statistics

Metabolite identification was performed through the use of several databases including PubChem Compound (Kim et al., 2016), KEGG (Kanehisa et al., 2016), and HMDB (Wishart et al., 2007, 2009, 2013) and the online chemical translation service (Wohlgemuth et al., 2010). Raw data were normalized to the average of pooled control data and results were plotted as in LOG2 scale, so that equal fold changes (up/down-regulated) will have the same distance to the zero baseline. For biomarker discovery, the linear SVM classification method was used with the feature ranking method UNIV AUROC. Univariate analysis was used to visualize the data because it is one of the most common methods utilized for exploratory data analysis and provides a preliminary overview about features that are potentially significant in discriminating the conditions under study. The Receiver Operating Characteristic (ROC) curve analysis is usually the method of choice of biomarker identification and performance evaluation because it provides a complete and easily visualized sensitivity/specificity report visualization. In a ROC curve, the true positive rate (Sensitivity) is plotted as function of the false positive rate (100-Specificity) for different cut-off points of a given parameter. Each point on the ROC curve represents a sensitivity/specificity pair corresponding to a particular decision threshold. A test with perfect discrimination (no overlap in the two distributions) has a ROC curve that passes through the upper left corner (100% sensitivity, 100% specificity). Therefore the closer the ROC curve is to the upper left corner, the higher the overall accuracy of the test, the closer the ROC curve to the diagonal line, the poorer the diagnostic power of the test. The area under the ROC curve is an index of how well a parameter can distinguish between two diagnostic groups (carrier/normal). In the Youden’s approach, the optimal cut-off is the threshold that maximizes the distance to the diagonal line defined by max (sensitivity + specificity). This analysis was performed with MetaboAnalyst (Xia et al., 2015). All metabolites obtained by mass spectrometry were subjected to two rounds of analyses. First, a list of single metabolites (or their ratios) was identified by the algorithm under the biomarker discovery feature of MetaboAnalyst. From these, only those with an area under the curve (AUROC) of ≥0.8 and *p* < 0.05 were selected. The second round of filtering involved the selection of markers that had a false positive rate of ≤10% and a true positive rate of ≥80%.

## Results

### Identification of Plasma Biomarkers

**FIGURE 1:**
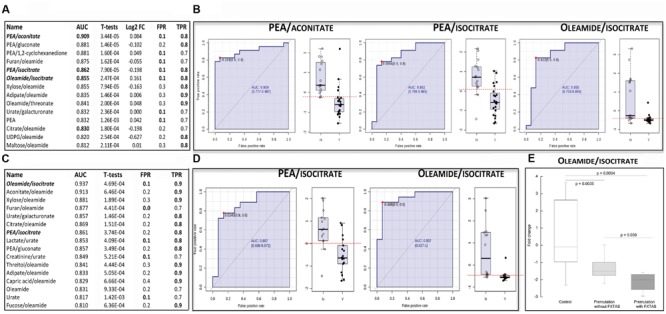
**Metabolites and their ratios as potential biomarkers of the premutation. (A)** The displayed biomarkers were obtained by using data from all controls and premutation carriers (reported in **Table 1**). The complete list of metabolites identified by mass spectrometry is reported in **Supplementary Table S1**. Metabolites’ performance was filtered based on their area under ROC curve (AUROC ≥ 0.800) and T-statistics (*p ≤* 0.05). Also shown are the generalized log transformation of the fold change (Log FC), false positive rate (FPR) and true positive rate (TPR). In bold, biomarkers with FPR ≤ 0.1 and TPR ≥ 0.8. **(B)** Receiver operator characteristic (ROC) curves of the model using metabolite ratios identified under A as potential biomarkers. The X-axis represents the FPR (as 1-specificity) whereas the Y-axis represents the sensitivity. On the right side of each ROC, box whisker plots of the generalized log transformation of fold change ratios in control (N) and premutation carriers (Y). **(C)** As in A but analysis performed with subjects not taking SSRIs (those excluded were indicated with asterisks under **Table 1**). **(D)** ROC curves of the model using metabolite ratios identified under C as potential biomarkers (other details under B). **(E)** Differences in the plasma biomarker oleamide/isocitrate in controls and carriers of the premutation with and without FXTAS. Data are shown as fold change and were analyzed by ANOVA followed by Bonferroni’s *post-hoc* test.

A total of 143 metabolites (**Supplementary Table S1**) were identified by mass spectrometry in plasma samples from 23 carriers of the premutation and 16 age- and sex-matched controls (see demographics details of the subjects under **Table 1**). The metabolites and their relative concentrations were analyzed by using a linear SVM classification method with the univariate AUROC feature ranking method. From this analysis, a list of single metabolites (or their ratios) was identified as being potential biomarkers of the premutation, from which only those with an AUROC of ≥0.8 and *p*-values ≤ 0.05 were selected (**Figure 1A**). These metabolites were classified based on their biological role (based on the KEGG BRITE functional hierarchy) as follows: biogenic amines (phenylethylamine; PEA), bioactive fatty acids (oleamide), organic carboxylic acids (dicarboxylic such as adipate and tricarboxylic such as citrate, aconitate, and isocitrate), monosaccharides (aldoses such as xylose and sugar acids such as glucuronic and galacturonic acids), and others (uric acid, furan, threonic acid, 1,2-cyclohexadione, uridine diphosphate glucuronic acid, or UDPG). The biomarkers that differentiated the most between the two diagnostic groups were oleamide ratios (eight instances), PEA alone or in ratios (five instances), followed by Krebs’ cycle intermediates (four instances; **Figure 1A**). By filtering the metabolites or their ratios shown under **Figure 1A** by a false positive rate of ≤10% and a true positive rate of ≥80%, the ratios of PEA/aconitate, PEA/isocitrate, and oleamide/isocitrate were the only ones fulfilling these thresholds (**Figure 1A**, shown in bold and italics). These metabolite ratios were then visualized by using the receiver-operator characteristic (ROC) curve analysis (**Figure 1B**).

Within these identified biomarkers, the plasma levels of two bioactive molecules, namely PEA and oleamide, in carriers of the premutation were lower than controls, whereas those of two Krebs’ cycle intermediates, aconitate, and isocitrate were higher (**Table 2**).

**Table 2:** Levels of metabolites identified as potential biomarkers of the premutation.

Metabolite	Log2 FC	-log10 (p)	FDR
Aconitate	0.5	2.77	0.111
Isocitrate	0.3	1.29	0.427
PEA	-0.6	3.49	0.046
Oleamide	-2.9	2.05	0.211

### Nutritional Supplements and Medications as Confounding Factors for Biomarker Validation

While the diagnostic groups were not different in terms of age or sex (see Materials and Methods), it could be argued that other confounding factors could influence certain metabolite variations, such as intake of vitamins, minerals, nutritional supplements, and medications. Participants on prescription medications were not excluded from this study. However, careful record of all prescription medications was kept. Of the subjects included in this study, 6 controls and 13 carriers were on multivitamins/probiotics or nutritional supplements, one control and eight carriers were on antidepressants, three controls and four carriers on cyclooxygenase inhibitors, two controls and two carriers on hormone replacement therapy, one control and two carriers on antihistamines, one control and one carrier on nitric-oxide producing drugs. Other medications included hydroxymethylglutaryl-CoA reductase inhibitors (two carriers), proton pump inhibitors (two carriers), beta2 agonist (two carriers), levodopa (one carrier), alpha2A receptor agonist (one carrier), alpha1 adrenergic blocker (one control), ACE inhibitor (one carrier), anticoagulant (one control), barbiturate (one carrier), beta blocker (one carrier), and inhibitor of monoamine transport (one carrier).

Carriers, although not significant, were more likely to take vitamins and supplements than controls (56.5% vs. 37.5%; *p* = 0.059). From the variety of medications that both groups were receiving (see Materials and Methods), carriers were more likely to be taking antidepressants than controls (30.4% vs. 6.25%; *p* < 0.0001). The most common class of antidepressant was constituted by the selective serotonin reuptake inhibitors (SSRI; *n* = 6, including one control), followed by serotonin–norepinephrine reuptake inhibitors (*n* = 2 carriers), serotonin modulators (*n* = 1 carrier), and dopamine–norepinephrine reuptake inhibitors (*n* = 1 carrier). While a careful review of the literature and known modes of action of these drugs does not suggest that these classes of supplements or medications are likely to influence any of the metabolites and/or biochemical pathways discussed in this study, we could not ascertain whether the differences in plasma metabolomics between controls and carriers were the sole result of the presence of the *FMR1* premutation, or its combination with the therapeutic use of SSRI and/or metabolic consequences from SSRI-derived side effects. To solve this issue, the biomarker discovery analysis was repeated under identical conditions but excluding those subjects that were taking SSRIs (indicated with asterisks under **Table 1**), which represented a 6.25% of the controls and a 21.7% of premutation carriers. Consistent with the previous findings, no statistically significant differences were found between groups of SSRIs-free controls and premutation carriers in terms of age, sex, or FXTAS subjects (36 ± 12 vs. 41 ± 17, *p* = 0.304; 40% males vs. 44.5, χ^2^ test *p* = 0.8002), except on the CGG repeat expansion (32 ± 5 vs. 96 ± 35, *p* = 6.2 × 10^-8^).

The resulting metabolites or ratios were screened for AUROC ≥0.8 and *p*-values ≤ 0.05 (**Figure 1C**) and those with a false positive rate ≤10% and a true positive rate ≥80% were selected as potential biomarkers (**Figure 1C** indicated in bold italics and **Figure 1D**). The resulting metabolite ratios were PEA/isocitrate and oleamide/isocitrate, two of the three biomarkers identified before (compare **Figures 1B** vs. **1D**). This analysis indicated that the presence of subjects taking SSRIs (which constituted an overall ~18% of all subjects) did not influence significantly the biomarkers identified before.

Finally, we tested whether any of the three biomarkers identified above (**Figure 1B**) differentiated among the three diagnostic groups, namely control and premutation with and without FXTAS. Although only four carriers of the premutation exhibited FXTAS, a significant difference between this group and both controls and unaffected carriers was observed with one of the biomarkers, namely oleamide/isocitrate (**Figure 1E**). This ratio was 30% lower in FXTAS-affected compared to the non-affected group (*p* = 0.039).

## Discussion

To the best of our knowledge, this is the first study in which an untargeted serum metabolomic profiling approach combined with sequential metabolite ratio analysis has been applied to discriminate plasma biomarkers in plasma of premutation and FXTAS-carriers. Our results demonstrate that a panel of four core serum metabolites (PEA, oleamide, aconitate, and isocitrate) can be used for sensitive and specific diagnosis of the premutation with and without FXTAS, and one of these ratios (namely, oleamide/isocitrate) as a biomarker of FXTAS. The findings of this study are promising because, despite the relatively small sample size (*n* = 16 and 23, respectively, for control and premutation individuals), they were obtained from a clinically well-characterized cohort of subjects with the *FMR1* premutation representing the wider clinical spectrum of this genetic disorder. Although it could be argued that plasma metabolites may not be predictive of those in CNS because the blood-brain barrier serves to limit the passage of metabolites from and to the periphery, significant correlations have been reported for a number of key metabolites between plasma and CSF in subjects with HIV-1 infection [among them lactate, glutamine, and citrate (Maher et al., 2011)]. Furthermore, several reports have applied metabolomics of non-invasive samples to study complex neurological disorders (Griffin and Salek, 2007) including schizophrenia and Parkinson’s disease (Dumas and Davidovic, 2013). Supporting the notion that plasma metabolites can be used as surrogates of brain function, blood plasma metabolic profiles (including lactate) consistent with metabolic syndrome and increased inflammation have been reported in elderly people with mild cognitive impairment, a transitional state with considerably increased risk for Alzheimer’s disease (Tukiainen et al., 2008), and high CSF citrate levels have been found associated with depression but not to manic features (Mellerup and Rafaelsen, 1981). If these correlations were also applicable to our study, the higher plasma lactate (**Figure 1C**) and citrate (**Figures 1A,C**) levels observed in carriers could be linked to their higher incidence of cognitive impairment (Grigsby et al., 2006, 2008; Brega et al., 2008) and depression (Bacalman et al., 2006).

Considering that a significant number of metabolites identified as potential biomarkers in this study are of mitochondrial origin or involved in mitochondrial metabolism (citrate, aconitate, isocitrate) and given the roles of mitochondria at providing ATP to aerobic tissues—such as brain—and on *de novo* synthesis of Krebs cycle-associated neurotransmitters [i.e., Glu, Asp, and GABA (Butterworth and Heroux, 1989; Laughlin et al., 1998; Attwell and Laughlin, 2001; Navarro et al., 2008; Napoli et al., 2014)], it is tempting to propose that these metabolites are pointing at an underlying mitochondrial dysfunction. Consistent with this view, we have reported deficits in bioenergetics and/or mitochondrial morphology and dynamics in fibroblasts (Ross-Inta et al., 2010; Napoli et al., 2011, 2016c) from carriers as well as in brains (Napoli et al., 2016a) and ovaries (Conca Dioguardi et al., 2016) from a murine model of the premutation. Methods used for assessing mitochondrial dysfunction are associated with significant drawbacks that limit their routine clinical use. Some of them are invasive (requiring a skeletal muscle biopsy), whereas non-invasive ones require specialized exercise physiology evaluation or NMR studies, which may not be widely available, and not practical for monitoring disease progression or therapeutic responses for subjects with limited mobility. Levels of key plasma metabolites (lactate, alanine, and pyruvate) are often suggestive of respiratory chain diseases or mitochondrial dysfunction (Robinson et al., 1986; Gillis and Kaye, 2002), but simply lack sensitivity and specificity [61% sensitivity based on (Scaglia et al., 2004)]. In this study, by applying a global metabolite profiling utilizing a relatively non-invasive method, a combination of metabolites was identified that have the potential to provide a sensitive and specific assessment of the mitochondria status or “fitness” in carriers, not influenced by SSRI treatments. In this context, it is important to point out that beneficial effects on mitochondrial function have been inferred from various studies based on the fact that anti-depressant treatments normalize decreased cerebral blood flow and glucose metabolism in the prefrontal cortex of depressed patients (Buchsbaum et al., 1997; Kennedy et al., 2001; Dimatelis et al., 2013). However, in our study, the same mitochondrial metabolites with and without SSRIs treatments were identified (citrate, aconitate, isocitrate) with the exception of lactate, which was noted in the SSRI-free group, possibly suggesting a marginal improvement provided by this therapy or that carriers do not respond positively to this pharmacological intervention.

Within these identified biomarkers, the plasma levels of PEA and oleamide were lower than controls, whereas those of two Krebs’ cycle intermediates, aconitate, and isocitrate were higher (**Table 2**). To understand the biological meaning of these biomarkers and their ratios, we focused on these metabolites and the pathophysiology behind them.

*Phenylethylamine-* Decarboxylation of Phe in dopaminergic neurons of the nigrostriatal system (Barroso and Rodriguez, 1996) result in the generation of PEA, the latter being a precursor to the neurotransmitter phenylethanolamine. However, some studies have indicated that PEA *per se* may function as a neuromodulator or neurotransmitter, stimulating the release of dopamine from the cytoplasmic pool and behaving as a dopamine receptor agonist (Raiteri et al., 1977; Barroso and Rodriguez, 1996). High levels of PEA have been found in the urine (but not in serum or CSF) of schizophrenics (Potkin et al., 1980; O’Reilly and Davis, 1994). Low levels—as those observed in carriers—have been reported in the CSF of subjects affected with Rett’s syndrome (Satoi et al., 2000) and in urine from Parkinson’s disease affected individuals (Zhou et al., 1997), children with attention deficit hyperactivity disorder (Kusaga et al., 2002) and subjects affected with depression (Sabelli et al., 1983). Some of these clinical features are shared by carriers of the premutation (Parkinsonism, ADHD, depression, autism; (Bacalman et al., 2006; Farzin et al., 2006; Berry-Kravis et al., 2007). Interestingly, the concentration of PEA or its metabolite phenylacetic acid have been proposed as useful biochemical markers in psychiatric and behavioral research (Davis and Boulton, 1994). This may also be true in neurodegenerative diseases, as a significant negative correlation between CSF concentration of PEA and severity of Parkinson’s disease (Hoehn and Yahr stage) has been reported (Zhou et al., 1997).

The observed lower PEA concentrations in carriers than controls may reflect any of these possibilities:

(1) PEA may be decreased due to an incipient neuronal degeneration as the amine is synthesized by the nigral dopaminergic neurons. These neurons could be decreased in carriers as loss of *FMR1* is associated with reduced numbers of dopaminergic neurons in the substantia nigra pars compacta (Fish et al., 2013) as it has also been observed in Parkinson’s disease (Morris et al., 1989; Colloby et al., 2012). Consistent with this view, Greenshaw et al. (1986) have found that degeneration of the substantia nigra results in depletion of dopamine (DA) and a decrease in the rate of PEA accumulation in deprenyl-treated rats.
(ii) Postsynaptic PEA catabolism may be increased in the premutation such as increased monoamine oxidase B (MAO-B) activity would be enough to accelerate the catabolism of PEA. Truncating mutations in MAO-B, such as those observed in individuals with schizophrenia—but not with autism—may result in a gain-of-function of activity (Piton et al., 2011). However, no genomic or functional data on MAO-B activity are available for this cohort of subjects.
(iii) Increased presynaptic dopamine concentrations are known to inhibit PEA synthesis at the decarboxylation step (Boulton et al., 1990), a phenomenon which may be relevant in carriers with Parkinsonism under treatment with levodopa (Hall et al., 2006). However, in our cohort of carriers (*n* = 23), only one subject presented Parkinsonism and was treated with levodopa.

In the context of the premutation, the lower plasma PEA concentrations may reflect either incipient nigrostriatal degeneration in subjects without yet significant signs of Parkinsonism or a reduced modulatory effect of PEA on a postsynaptic receptor in response to dopamine.

*Oleamide-* Oleamide is a natural occurring fatty amide that accumulates in the CSF during sleep deprivation and induces sleep in animals. In this regard, it is being studied as a potential medical treatment for mood and sleep disorders, and cannabinoid-regulated depression. The mechanism of action of oleamide’s sleep inducing effects is not well understood yet; however, it is likely that this compound interacts with multiple neurotransmitter systems. Oleamide is structurally related to the endogenous cannabinoid anandamide. Both anandamide and oleamide elicit behavioral effects indicative of cannabinoid activity, but only anandamide binds the cannabinoid (CB1) receptor *in vitro*. Oleamide, anandamide, and myristic amide are degraded to the corresponding fatty acids (oleic acid, arachidonic acid, and myristic acid) by the enzyme fatty-acid amide hydrolase (FAAH) to terminate the signaling functions of these molecules (Wei et al., 2006). This enzyme is most abundant in neocortex, hippocampal formation, amygdala, and cerebellum suggesting that this CNS distribution supports the degradation of neuromodulatory fatty acid amides at their sites of action influencing their effects on sleep, euphoria, and analgesia (Thomas et al., 1997).

Oleamide has been proposed to induce its behavioral effects by serving as a competitive substrate for the brain FAAH and inhibiting the degradation of endogenous anandamide. Disruption of the endocannabinoid pathway induces metabolic imbalances (Di Marzo et al., 2001; Osei-Hyiaman et al., 2005, 2006), outcomes generally considered detrimental to any tissue but, they gain more weight in brain given its high aerobic capacity (Laughlin et al., 1998; Attwell and Laughlin, 2001) and the localization of CB1 receptors in neurons and neuronal mitochondria (Benard et al., 2012). In FAAH^+/+^ and FAAH^-/-^ mice, oleamide induced hypomotility, hypothermia, and ptosis, all of which were enhanced in FAAH^-/-^ mice, with negligible binding to the CB1 receptor in brain extracts from either genotype (Lichtman et al., 2002). In contrast, anandamide exhibited a 15-fold increase in apparent affinity for the CB1 receptor in brains from FAAH^-/-^ mice, consistent with its pronounced CB1-dependent behavioral effects in these animals. Other reports indicated that oleamide neither directly activates CB1 receptors nor acts via the proposed “entourage” effect (Mechoulam et al., 1997) to increase concentrations of anandamide through FAAH inhibition, rather the selective effects of oleamide on theta-burst-conditioning may reflect modulation of GABAergic transmission (Lees and Dougalis, 2004). It has been proposed that genetic variations in FAAH can be associated with susceptibility to polysubstance abuse (OMIM: 606581). Indeed, a homozygous T129 variant is strongly associated with drug and alcohol abuse and methamphetamine dependence (Sipe et al., 2002; Filbey et al., 2010; Buhler et al., 2014) whereas increasing amygdala anandamide (and possibly oleamide) enables extinction-driven reductions in fear in mouse and may promote stress-coping in humans (Gunduz-Cinar et al., 2013).

Based on these reports and in the context of the premutation, lower levels of oleamide may be predictive or linked to the increased incidence of substance abuse, including alcohol (Kogan et al., 2008), mood/anxiety issues of carriers of the premutation (Kogan et al., 2008), their increased anxiety as a reflection of lower stress-coping abilities (Bourgeois et al., 2007) and a reflection of their sleep disturbance which is seen in the majority of carriers (Chonchaiya et al., 2010).

*Krebs’ cycle intermediates: citrate, isocitrate and aconitate*- Two intermediates of the TCA cycle, located within the first half of the cycle, namely aconitate and isocitrate, were identified as potential biomarkers along with citrate (**Figures 1A,B**). Their levels were higher in plasma from carriers than controls (1.4-fold of controls for aconitate and 1.2-fold for both citrate and isocitrate; *p* < 0.05). Increases in these tricarboxylic acids located within the first half of the cycle may indicate a slower Krebs’ cycle activity. This is based on the fact that increases in succinate, malate, and fumarate (located in the second half of the cycle) have been documented to accompany exercise, effect termed as the TCA cycle expansion (Gibala et al., 1997a,b). The “expansion” is believed to result from a relative excess of glycolysis compared to OXPHOS, which then results in the shunting of pyruvate and Glu to Ala and alpha-ketoglutarate (AKG) via Ala aminotransferase. As a result, AKG is then converted to other TCA cycle intermediates (succinate, malate, and fumarate). Thus, increases in the first half of this cycle are suggestive of lower alpha-ketoglutarate dehydrogenase (AKGDH) activity. This would result in higher AKG concentrations, which via the near-equilibrium of glutamate dehydrogenase, results in the increase of Glu concentrations. The latter is an allosteric inhibitor of glutaminase, and AKG is also an allosteric inhibitor of mitochondrial transport of Gln. Hence, an increase in AKG, due to a decreased activity of AKGDH, should lead to increases in Glu and Gln, and given that Glu is a precursor of GABA, also an increased synthesis of GABA or related metabolites. Two experimental evidences supported this option suggesting an increased flux from AKG to Gln: (i) the ratio of Gln-to-Glu was higher in plasma of premutation than controls (0.86 vs. 0.37; *p* < 0.05) and (ii) higher 4-hydroxybutyrate (GHB, a GABA derivative) was observed in plasma from carriers (twofold; *p* < 0.05) suggesting an increased synthesis of GABA from Glu and, as a result, of its product GHB.

In the context of the full and premutation, dysregulation of neurotransmitter systems, including the mGluR1/5 pathway and GABA pathways, has been reported in fragile X syndrome (Hagerman et al., 2010), neurons from a KI mouse model of the premutation (Cao et al., 2012) and in the brain of individuals with FXTAS (Pretto et al., 2014). Although not pathognomonic, the increased plasma levels of three TCA cycle metabolites (citrate, aconitate, and isocitrate) along with a higher plasma lactate (1.5-fold of controls; *p* = 0.095) have been observed in some mitochondrial diseases (Crippa et al., 2015), usually associated with the formation of analogs of TCA intermediates with the potential of depleting oxaloacetate and slowing down the TCA cycle, especially if not accompanied by the activation of the anaplerotic reaction catalyzed by pyruvate carboxylase. These results are consistent with the reports on mitochondrial dysfunction and abnormal network/distribution in cells from premutation carriers (Ross-Inta et al., 2010; Napoli et al., 2011, 2016c) as well as in KI mouse models of the premutation (Kaplan et al., 2012; Conca Dioguardi et al., 2016; Napoli et al., 2016a).

In the broader context, this and other studies (Fonteh et al., 2007; Griffin and Salek, 2007; Dumas and Davidovic, 2013; Tsuruoka et al., 2013; Napoli et al., 2015) highlight the potential of metabolic phenotyping to (a) identify biomarkers of neurological diseases utilizing non-invasive samples such as plasma and urine, (b) characterize metabolic profiles that contribute to accurate diagnosis in complex neurological conditions (endophenotypes), and (c) assess treatment efficacy and determine metabolic secondary side effects, especially in subjects with multidrug treatments associated with metabolic disorders.

Further studies are warranted to allow the construction of robust models for predicting neurological changes from the less invasive approach of plasma metabolic profiling and verifying the important biological roles of these key metabolites with endophenotypes (e.g., with and without Parkinsonism). If successful, the specific biochemical fingerprints could eventually be used to identifying key pathways for therapy and helping in monitoring disease progression. In this regard, the biomarker oleamide/isocitrate seemed to discriminate carriers with FXTAS from those not affected; however, future research would need to be performed to test whether this ratio can be used to diagnose more accurately stages of FXTAS as well as if it has any predictive value in terms of discriminating carriers that will develop FXTAS later in life.

## Author Contributions

CG conceptualized and designed the study, wrote the manuscript, and approved the final manuscript as submitted; EN separated the plasma from samples, contributed to the writing of the manuscript, revised and approved the final version as submitted; FT provided the CGG repeat sizing, revised the manuscript and approved the final manuscript as submitted; JH helped with the collection and classification of nutritional supplements and medications for the study group, revised and approved the manuscript as submitted; RH carried out clinical assessment of these subjects, wrote clinical findings, revised the manuscript and approved the final manuscript as submitted.

## Conflict of Interest Statement

RH has received funding from Novartis, Roche/Genentech, Alcobra, and Neuren for treatment trials in fragile X syndrome, autism and Down syndrome. She has also consulted with Novartis, Zynerba and Roche/Genentech regarding treatment for fragile X syndrome. All the other authors declare that the research was conducted in the absence of any commercial or financial relationships that could be construed as a potential conflict of interest.

## Supplementary Material

TABLE S1 **Complete list of metabolites identified by mass spectrometry**. Metabolite identification was performed through the use of several databases including PubChem Compound, KEGG and HMDB, and the online chemical translation service, as described in the Methods.
